# Acetylation in Tumor Immune Evasion Regulation

**DOI:** 10.3389/fphar.2021.771588

**Published:** 2021-11-22

**Authors:** Jun Lu, Xiang He, Lijuan Zhang, Ran Zhang, Wenzheng Li

**Affiliations:** ^1^ Hunan Normal University School of Medicine, Changsha, China; ^2^ Key Laboratory of Molecular Radiation Oncology Hunan Province, Changsha, China; ^3^ Xiangya Cancer Center, Xiangya Hospital, Central South University, Changsha, China; ^4^ Department of Radiology, Xiangya Hospital, Central South University, Changsha, China

**Keywords:** acetylated modifications, tumor immunity, acetylases, deacetylases, cancer immunotherapy

## Abstract

Acetylation is considered as one of the most common types of epigenetic modifications, and aberrant histone acetylation modifications are associated with the pathological process of cancer through the regulation of oncogenes and tumor suppressors. Recent studies have shown that immune system function and tumor immunity can also be affected by acetylation modifications. A comprehensive understanding of the role of acetylation function in cancer is essential, which may help to develop new therapies to improve the prognosis of cancer patients. In this review, we mainly discussed the functions of acetylase and deacetylase in tumor, immune system and tumor immunity, and listed the information of drugs targeting these enzymes in tumor immunotherapy.

## Introduction

The main driving force for tumor initiation and progression are not only the alterations in cancer cells but also the influences of immune system and tumor immune microenvironments (Bindea et al., 2013; Jones et al., 2019 ). As an important hallmark of cancer, tumor cells evade immune surveillance by suppressing the immune system and having low immunogenicity (Peng et al., 2019; Gao et al., 2020). For example, aberrant expression of immune checkpoints (ICs) components, such as the programmed cell death protein 1 (PD-1), cytotoxic T lymphocyte-associated protein 4 (CTLA-4), T-cell immunoglobulin and mucin domain-containing lymphocyte activation gene 3 (LAG-3), and LAG-3 with Ig and ITIM structural domains T-cell immune receptors (TIGIT), creates an immune destructive environment that promotes tumor cells escape immune destructions (Saleh et al., 2020). *Cancer* immunotherapies such as cancer vaccines, adoptive T-cell therapy (ACT), and immune checkpoint blockade (ICB), which kills cancer cells through employing the body’s own immune system, have achieved encouraging progress within the last decade (Wang S. et al., 2019). Despite these breakthroughs, only 10–30% of patients respond to and benefit from them, and the underlying reasons of these low benefits are due to the development of primary and acquired drug resistance, low response frequency of some cancers, and the heterogeneity of tumors (Sharma et al., 2017). Therefore, the urgent question is how to enhance patient responsivity and benefit from immunotherapy by targeting tumor cells or modulating tumor immune microenvironment.

Recently, there is growing evidence showing that epigenetic modification is essential to regulation of tumor immunity and immunotherapy, which provides a possible target for improving the outcome of immunotherapy. Epigenetic gene regulation, which alters gene expression and function without involving alterations in DNA sequences, is an important regulatory process in cellular biology. Several types of epigenetic modification were identified such as DNA methylation, histone modification, miRNA regulation, genomic imprinting, and chromosome remodeling, among which acetylation is considered one of the most common types of epigenetic modification (Lawrence et al., 2016). Histone acetylation is mediated by histone acetyltransferase (HAT), while deacetylation is mediated by histone deacetylase (HDAC). Acetylation of histones alters the secondary structure of the histone tail, leading to relaxation of the chromatin structure by increasing the distance between DNA and histones, opening up tracts of DNA and allowing for increased binding of transcription factor complexes to gene promoter sequences, thereby upregulating transcription (Kouzarides, 2007; Lawrence et al., 2016). In contrast, histone deacetylation usually promotes chromatin condensation and down-regulates the transcriptional level of related genes, and is usually accompanied by an increase in histone methylation of the same residue.

Besides, there is growing evidence that abnormal histone acetylation modification is related to the pathological process of cancer through regulating the expression of oncogenes and tumor suppressors (Audia and Campbell, 2016 ). For instance, previous studies have shown that changes in histone acetylation level, especially the loss of acetylated Lys16 of histone H4, is related to the development of many cancers and is a common feature in human tumor cells (Fraga et al., 2005). Due to the critical role of acetylation in tumorigenesis, several drugs targeting HDAC have been developed to treat cancer. Furthermore, recent studies have shown acetylation modification is essential for immune system function and tumor immunity.

Therefore, in view of the effects of acetylation modification on tumor cells and immune system, as well as the clinical use of the HATs and HDACs inhibitors, it is worth exploring whether HATs and HDACs could influence immunotherapy efficacy by altering the tumor immune microenvironment through acetylation regulation on tumor cells or immune cells, and whether targeting these enzymes may improve the efficacy of immunotherapy. In this review, we focus on how HATs and HDACs modulate tumor immunity and discuss the potential application of drugs targeting these enzymes to improve the outcome of immunotherapy.

## HATs in Tumor Immunity Regulation

There are three major HAT subfamilies in human: the GCN5-related N-acetyltransferase (GNAT) subfamily including PCAF and GCN5; the MYST subfamily including TIP60, MOZ, MORF, MOF and HBO1; the p300 subfamily including p300, CBP. Besides these enzymes, HATs also include TAT1, ESCO1, ESCO2 and HAT1 (Narita et al., 2019). In addition to acetylated histones, HATs can also directly acetylate a range of transcription factors such as C/EBPα, FOXO1, and p53, resulting in the regulation of transcription factor activity and thereby regulation of cancer progression (Sheikh and Akhtar, 2019). At present, only a portion of HATs have been shown to be involved in tumor immunity regulation (Table 1).

**TABLE1:** Function of HATs related to tumor immunity in different cell types.

Protein	Cell type	Target	Function	References
PCAF	Tregs	Foxp3	acetylate Foxp3 to impair Tregs function	Liu et al. (2019c)
	Macrophages	TNF-α, IL-6 and CXCL10	inhibit the inflammatory response of M1 macrophages	Wang et al. (2021)
	CD4 and CD8 T Cells	Foxp3	reduce tumor volume and improves anti-tumor immunity	Liu et al. (2019c)
GCN5	Tregs	ISG	regulate the development of T regulatory cells and the transcription of ISG expression	Au-Yeung and Horvath, (2018)
	Tregs	Foxp3	*in vivo* deletion inhibit Treg and Teff cells function	Liu et al. (2019c)
	iNKT Cells	--	promote iNKT cells development	Wang et al. (2017)
	Head and Neck Squamous Cell Carcinoma	H3K27	activate transcription of PD-L1 and galectin-9 to evade tumor immunity	Ma et al. (2020)
p300/CBP	Tregs	Foxp3	protect the function of Foxp3+ Tregs and suppress anti-tumor immunity	Liu et al. (2013)
				Liu et al. (2014)
	T and B Cells	IL2 and IL10	promote Treg differentiation by inducing T and B cells to secrete the pro-inflammatory cytokines IL2 and IL10	Castillo et al. (2019)
	Immunosuppressive Cells	--	promote tumor progression by protecting the function of immunosuppressive cells in tumor microenvironments	Liu et al. (2013)
				de Almeida Nagata et al. (2019)
	Liver *Cancer*	MEF2D	induce PD-L1 expression in liver cancer by acetylation of MEF2D to impair CD8^+^ T cell-mediated anti-tumor immunity	Xiang et al. (2020)
				Guo et al. (2021)
	CTL	MHC-I	regulate tumor cell immunogenicity	Zhou et al. (2021)
Tip60	Tregs	Foxp3	promote FOXP3 mediated inhibition and T cell mediated suppression	Li et al. (2007)
	Tregs	Usp7	control Treg function and limit tumor progression	Wang et al. (2016)
HAT1	Pancreatic *Cancer*	--	transcriptional upregulation of PD-L1 in tumor cells	Fan et al. (2019)

Abbreviations: Treg, regulatory T cells; TNF, tumor necrosis factor; IL, interleukin; CXCL, C-X-C motif chemokine ligand; ISG, IFN-stimulated gene; NK, natural killer cell; CTL, cytotoxic T cell; MHC, major histocompatibility class; Usp, ubiquitin-specific protease.

**TABLE 2:** Function of HADCs related to tumor immunity in different cell types.

Protein	Cell type	Target	Function	References
HDAC1	Gastric *Cancer*	STAT1	enhance the expression of JAK2, *p*-JAK1, *p*-JAK2, and p-STAT1 and promote the nucleus translocation of STAT1	Deng et al. (2018)
	Cervical *Cancer* and GBM	Keap1	repress membrane expression of MHCⅡ	Wijdeven et al. (2018)
	NSCLC	Sp1	repress the activity of sp1 to impair the membrane expression of cd1d	Yang et al. (2012)
	Macrophages	miR-146a	induces tumor associated macrophages to adopt the M1-like phenotype	Gao et al. (2015)
				Ji et al. (2019)
	Tregs	IL-2 and the IFN- *γ* promoter	participate in the formation of the CoRest which promote function of Treg	Xiong et al. (2020)
HDAC2	Multiple Cell Lines	PD-L1	deacetylate PD-L1 to promote the nuclear translocation of PD-L1 which enhance the expression of PD-L1 and MHC Ⅰ	Gao et al. (2020)
	Melanoma	ISG promoter	deacetylates IFN-stimulated genes (ISG) promoter at H4K16 to enhances the infiltration of tumor lymphocytes	Zhang et al. (2015)
	Macrophages	c-Jun promoter	repress the expression of c-Jun	Fang et al. (2018)
				Wu et al. (2019)
HDAC3	Melanoma	Runx3	inhibits the cytotoxicity of CD8+T cells and recognition ability of NK cells	Fiegler et al. (2013)
				Tay et al. (2020)
	Monocytes and Mø	--	inhibit LPS induced cytokine secretion	Ghiboub et al. (2020)
	Breast *Cancer* and Colorectal *Cancer*	--	down-regulate PD-L1 expression via HDAC3/p300-mediated NF-κB signaling	Lucas et al. (2018)
				Wang et al. (2020a)
	B-cell Lymphoma	PD-L1 promoter	repress PD-L1 expression via being recruited to the PD-L1 promoter by the transcriptional inhibitor BCL6	Deng et al. (2019)
	Pancreatic *Cancer*	--	enhance PD-L1 expression through STAT3 signaling pathway	Hu et al. (2019)
HDAC4	Brain Astrocytes	liver X receptor	HDAC4 impairs LXR-Induced suppression of STAT1 binding to promoters and downstream inflammatory gene expression	Lee et al. (2009)
	Cervical *Cancer*	IFN-α–stimulated promoter	promote type I interferon signaling via recruiting STAT2 to IFN-α–stimulated promoters	Kalbasi and Ribas, (2020)
HDAC5	CD4+T Cells	Foxp3	positively related to the transformation of Treg and the production of IFN *γ*	Xiao et al. (2016)
	Macrophages	MKL1	impair TNF-α induced pro inflammatory gene transcription	Li et al. (2017)
	Macrophages	SOCS3	recruit CCR2+ macrophages to promote macrophages to reaggregate into tumor	Hou et al. (2020)
HDAC6	APCs	IL-10 promoter	recruit to immunosuppressive cytokine IL-10 promoter with STAT3	Cheng et al. (2014b)
	Macrophages	--	inhibit the expression of IFN *γ* and IL-2	Knox et al. (2019b)
	GBM and Melanoma	STAT3	promote the recruitment and activation of STAT3 to enhance the expression of PD-L1	M et al. (2016)
				Liu et al. (2019a)
	HCC	Foxo1	reduce the expression of PD-L1 and inhibit Foxo1 nuclear translocation to limit TH17 pathogenicity	Qiu et al. (2020)
	Melanoma	--	up-regulate the expression of tumor-associated antigens and MHC-I	Woan et al. (2015)
HDAC7	Pre-B Cells	MEF2C	repress transdifferentiation of Pre-B cells into macrophages	Barneda-Zahonero et al. (2013)
	CD4 T Cells	Nur77 and Irf4	repress the IFN-γ production and CD4^+^ T cells proliferation	Myers et al. (2017)
	Macrophages	PKM2	deacetylate PKM2 to increase the expression of IL-1β	Das Gupta et al. (2020)
HDAC8	Melanoma	PD-L1 promoter	prevent transcription factors to bind to PD-L1 promoter	Wang et al. (2018b)
	HCC	--	repress intra tumoral CD8+T cell infiltration	Yang et al. (2021e)
	Keratinocytes	--	increases proinflammatory gene expression	Sanford et al. (2016)
HDAC9	CCRC	--	activates immune cell infiltration and increase expression of immunological molecules such as PD-L1, CTLA4 and LAG3	Fu et al. (2020b)
	NSCLC	--	promote the CD8+DC infiltration of the TME and DC antigen presentation	Ning et al. (2020)
	Macrophages	TBK1	enhance the kinase activity of TBK1 to activate antivirus innate immunity	Li et al. (2016b)
HDAC10	NSCLC	--	positively correlated with the expression of PD-L1	Liu et al. (2020c)
	HCC	CXCL10 promoter	recruit EZH2 to CXCL10 promoter to inhibit CXCL10 expression thus repressing NK cell migration	Bugide et al. (2021)
HDAC11	APC	IL-10 promoter	interacts with the IL-10 promoter to repress IL-10 expression	Villagra et al. (2009)
	MDSCs	C/EBPβ promoter	repress expression of C/EBPβ to inhibit immunosuppressive arginase and iNOS	Chen et al. (2021)
	T Cells	Eomesodermin and Tbx21 promoter	repress transcription factors Eomesodermin and Tbx21 to reduce the production of inflammatory cytokine and effector molecule	Woods et al. (2017)
Sirt1	Th1/17 Cells	Foxo1	enhance the activity of Foxo1 to promotes the expression of Klf2 and Ccr7 genes	Chatterjee et al. (2018)
	Tregs	Foxp3	promote the proteasomal degradation of Foxp3 via deacetylating Foxp3 to repress Treg activation	van Loosdregt et al. (2010)
	Mesenchymal Stem Cells	p65	deacetylate p65 to reduce the expression of iNOS which impair the immunosuppressive ability of MSCs	Zhou et al. (2019)
	Mesenchymal Stem Cells	--	induce IFN *γ* and CXCL10 expression to recruit NK cells	Yu et al. (2016b)
				Ye et al. (2020)
	NSCLC	Snail	deacetylate and stabilize transcriptional factor Snail to inhibits transcription of Axin2 which leads to enhanced binding of β-catenin/TCF to PD-L1 promoter	Yu et al. (2016a)
	HCC	--	promoted M1 macrophage polarization via NF-κB signaling	Chen et al. (2015)
Sirt2	T Cells	GSK3 β	promote aerobic oxidation in CD8+T and differentiation of CD8+T cells into TEM	Jiang et al. (2020)
	NK Cells	--	promotes Erk1/2 and p38 MAPK signaling in activated NK cells	Chen et al. (2019a)
	Multiple Malignant Myeloid Cells	CDK9	enhance IFN signaling via deacetylating CDK9 which promote STAT1 phosphorylation at Ser-727	Kosciuczuk et al. (2019)
Sirt3	Prostate *Cancer*	--	reduce the infiltration of CD4^+^ T cells, macrophages and neutrophils via decreasing the level of CCL8 and CXCL2	Fu et al. (2020a)
	Mø	NLRC4	deacetylates NLRC4 to promote its activation which promotes inflammasome activation	Guan et al. (2021)
Sirt4	Tregs	--	inhibit FoxP3, anti-inflammatory cytokines IL-10, TGFβ and AMPK signaling to impair the anti-inflammatory function of Treg	Lin et al. (2019)
Sirt5	CCRC	--	promote immune cell infiltration	Lu et al. (2021)
Sirt6	Breast *Cancer*	IκB	deacetylate IκB to suppresses the expression of PD-L1	Song et al. (2020)
	Pancreatic *Cancer*	--	increased the production of TNF and IL8 which leads to pro-inflammatory and pro-angiogenic phenotype	Vanamee and Faustman, (2017)
Sirt7	Breast *Cancer*	--	positively correlated with the expression of IRF5 and PD1, M1 macrophages and depletes T cells	Li et al. (2016a)
	HCC	MEF2D	deacetylate MEF2D to inhibit its binding to PD-L1 promoter	Castillo et al. (2019)

Abbreviations: STAT, signal transducer and activator of transcription; JAK, janus kinase; NSCLC, Non-small-cell carcinoma; Treg, Regulatory T cell; IL, interleukin; IFN, interferon; PD-L1, programmed death-ligand 1; ISG, IFN-stimulated gene; LPS, lipopolysaccharides; NF-κB, nucleus factor κ-light-chain enhancer of activated B cells; PKM, pyruvate kinase M2; CTLA, cytotoxic T lymphocyte-associated protein; LAG, lymphocyte activation gene; Foxo, forkhead box O; APCs, antigen-presenting cells.

**TABLE 3:** Information of drugs targeted to acetylase and deacetylase.

Drugs	Target enzymes	IC50	Clinical trials stage	References
E7386	--	--	Phase1	
PRI-724	--	--	Phase1-2	
A485	p300-BHC	9.8 nM	--	Lasko et al. (2017)
	CBP-BHC	2.6 nM	--	Lasko et al. (2017)
Panobinostat (LBH589)	HDAC1	2.5 nM	Phase1-4	Li et al. (2020a)
	HDAC2	13.2 nM		
	HDAC3	2.1 nM		
	HDAC4	203 nM		
	HDAC5	531 nM		
	HDAC7	531 nM		
	HDAC8	277 nM		
	HDAC9	5.7 nM		
Chidamide (CS055/HBI-8000)	HDACs	0.296 ± 0.0417 μM,112 ± 20 nM	Phase1-4	Lu et al. (2017)
				Chen et al. (2019b)
Trichostatin-A (TSA)	HDACs	0.0125 ± 0.0012 μM	Phase1-4	Chen et al. (2019b)
ACY738	HDACs	1.7 nM	--	Jochems et al. (2014)
Nexturastat A	--	--	--	
Mocetinostat (MGCD0103)	HDAC1	142 nM	Phase1-2	Atadja, (2009)
	HDAC2	147 nM		
	HDAC3	205 nM		
	HDAC4	>30000 nM		
	HDAC5	1889 nM		
	HDAC6	>30000 nM		
	HDAC7	>30000 nM		
	HDAC8	28167 nM		
	HDAC9	1177 nM		
	HDAC10	54.9 nM		
	HDAC11	104 nM		
Mocetinostat (MGCD0103)	HDACs	2.76 ± 1.98 μM	Phase1-2	Gorshkov et al. (2019)
Domatinostat (4SC-202)	--	--	Phase1-2	
Valproic acid (VPA)	HDAC1	0.7–1 mM	Phase1-4	Gurvich et al. (2004)
	HDAC2	0.7–1 mM		
	HDAC3	0.7–1 mM		
	HDAC4	1–1.5 mM		
	HDAC5	1–1.5 mM		
	HDAC7	1–1.5 mM		
Tacedinaline (CI994)	--	--	Phase2-3	
CXD101	HDAC1	63 nM	Phase1-2	Eyre et al. (2019)
	HDAC2	570 nM		
	HDAC3	550 nM		
AR42	--	--	Phase1	
Belinostat(PDX101)	HDAC1	17.6 nM	Phase1-2	Atadja, (2009)
	HDAC2	33.3 nM		
	HDAC3	21.1 nM		
	HDAC4	1,236 nM		
	HDAC5	76.3 nM		
	HDAC6	14.5 nM		
	HDAC7	598 nM		
	HDAC8	157 nM		
	HDAC9	44.2 nM		
	HDAC10	31.3 nM		
	HDAC11	44.2 nM		

Abbreviations: P300-BHC, E1A binding protein p300-bromodomain-HAT-C/H3; CBP-BHC, cyclic-AMP, response element binding protein-bromodomain-HAT-C/H3.

### P300/CBP-Associated Factor

P300/CBP-associated factor (PCAF), also named lysine acetyltransferase 2B (KAT2B) (Liu T. et al., 2019), is a HAT that mainly acetylates H3 histones, as well as a number of non-histone proteins that coordinate carcinogenic and tumor suppressive processes (Wang LT. et al., 2020). Previous studies have shown that the expression of PCAF is reduced as a tumor suppressor in esophageal, breast, ovarian, colorectal and pancreatic cancers, and that loss of PCAF expression is associated with poor prognosis in gastric cancer and may serve as a potential biomarker for invasive and aggressive tumors (Brasacchio et al., 2018). For example, it acts as a suppressor of HCC progression by promoting apoptosis through acetylation of glioma-associated oncogene homolog-1 (Gli1), histone H4 and the phosphatase and tensin homolog deleted on chromosome 10 (PTEN). On the contrary, PCAF was reported to be highly expressed in HCCs and to promote tumor progression via acetylation of phosphoglycerate kinase 1 (PGK1), pyruvate kinase M2 (PKM2), and glyceraldehyde-3-phosphate dehydrogenase (GAPDH), which subsequentially induces the Warburg effect and activates Akt signaling (Zheng et al., 2013; Wang T. et al., 2018).

With its modulatory effects on tumors, PCAF can also regulate immune system function. In a study of Foxp3+ Treg cells, PCAF was found to contribute to the inhibition of Treg cells apoptosis in response to TCR stimulation and to increase inducible Tregs (iTregs) production via IL-2 and TGF-β. In addition, PCAF acetylates Foxp3 to impair Treg cells function (Liu Y. et al., 2019). Another study has shown that overexpression of PCAF significantly suppressed the expression of pro-inflammatory genes TNF-α, IL-6 and CXCL10, suggesting that PCAF is a potential negative regulator of the inflammatory response of M1 macrophages (Wang et al., 2021).

There is limited evidence reporting the role of PCAF in tumor immunity regulation. A study reported that in lung adenocarcinoma tumor growth was compromised in PCAF^−/−^ mice, with reduced infiltration of CD4+Foxp3+ Treg cells but increased infiltration of host CD8 T cells, indicating that targeting PCAF reduces tumor volume and improves anti-tumor immunity (Liu Y. et al., 2019). More research on the role of PACF in tumor immunity in other cancer types is needed in the future.

### GCN5

General control non-depressible 5 (GCN5) mainly responsible for acetylation of H3K27, is the first histone acetyltransferase to be characterized in saccharomyces cerevisiae. GCN5 is highly expressed in a variety of human cancers and promotes cancer progression by participating in the acetylation of many non-histone proteins (Haque et al., 2021). An example is in prostate cancer, where upregulated GCN5 downregulates Egr-1 expression via the PI3K/PTEN/Akt signaling pathway, negatively affecting IL-6-induced prostate cancer cell metastasis and epithelial-mesenchymal transition (EMT) (Shao et al., 2018).

In the immune system, GCN5 was reported to regulate the development of Treg cells and the transcription of interferon (IFN)-stimulated gene (ISG) expression (Au-Yeung and Horvath, 2018). In Foxp3+ Treg cells, GCN5 deletion has no effect on Treg *in vitro* but inhibits Treg function *in vivo*, and impairs T-effector (Teff) cells function (Liu Y. et al., 2019). For invariant natural killer T (iNKT) cells, the deficiency of GCN5 blocks its development (Wang et al., 2017).

At present, studies on the role of GCN5 in tumor immunity are still relatively few. Overexpression of GCN5 and PCAF in solid tumors *in vivo* enhances immune surveillance and associated NKG2D-dependent tumor cell death (Hu et al., 2021). Knockdown of GCN5 and PCAF in osteosarcoma and lung cancer resulted impaired induction of the natural killer group 2D (NKG2D) ligand Rae-1 by IL-12 and the chemotherapeutic agent doxorubicin as inhibition of NKG2D ligand expression was associated with tumor cell death and accelerated tumor progression (Hu et al., 2017). On the contrary, in head and neck squamous cell carcinoma, GCN5 acetylates H3K27, which activates transcription of PD-L1 and galectin-9 to evade tumor immunity (Ma et al., 2020). More research is needed to explain this opposite trend and to provide more precise strategies for cancer therapy.

### p300/CBP

p300 (E1A binding protein p300) and CBP[CREB] (cyclic-AMP response element binding protein) binding proteins are considered functional homologs, sharing 63% homology at the amino acid level and exhibiting high structural similarity and functional redundancy (Ogryzko et al., 1996; Iyer et al., 2004; He et al., 2021). p300/CBP as a vital transcriptional co-activator and HAT contributes to a variety of cellular activities and plays a role in immune-mediated diseases and cancers through chromatin remodeling and gene activation (Karamouzis et al., 2007; Dancy and Cole, 2015). Overexpression or mutations of p300/CBP are found in malignant tumor, such as prostate and breast cancers (Bouchal et al., 2011; Hickey et al., 2021).

Previous studies have found that p300 and CBP are essential for the development and function of Foxp3+ Treg cells. Inhibition of p300 and CBP impairs Foxp3+ Treg cells function and furthers antitumor immunity (Liu et al., 2013; Liu et al., 2014). p300/CBP promotes Treg differentiation by inducing T and B cells to secrete the pro-inflammatory cytokines IL2 and IL10 (Castillo et al., 2019). It was observed that Treg was reduced in follicular lymphoma in tissues carrying CBP/p300 loss-of-function mutations (Castillo et al., 2019). In breast and lung cancers, p300/CBP inhibition can restrict tumor progression by impairing the function of immunosuppressive cells such as regulatory T cells and MDSCs in tumor microenvironments (Liu et al., 2013; de Almeida Nagata et al., 2019).

Several studies reported p300 can induce the expression of PD-L1 in liver cancer to impair CD8^+^ T cell-mediated anti-tumor immunity (Xiang et al., 2020; Guo et al., 2021), probably via acetylation of myocyte enhancer factor 2D (MEF2D). Furthermore, targeting p300/CBP by small molecular inhibitors such as E7386 and A485 could remarkably enhance the efficacy of PD-L1 blockade therapy in prostate and breast cancers in preclinical mouse models (Liu J. et al., 2020; Yamada et al., 2021). Additionally, p300 has been reported to regulate tumor cell immunogenicity, p300 ablations prevent chemotherapy-induced processing and presentation of major histocompatibility class I (MHC-I) antigens and abrogating the rejection of low MHC-I-expressing tumors by reinvigorated CD8 cytotoxic T cells (CTLs) (Zhou et al., 2021).

### Tip60

Tat-interactive protein 60-kDa (Tip60, also known as KAT5) is one of the MYST subfamily of HATs and was originally identified as a tat-interacting protein widely involved in DNA damage repair, cellular activity and carcinogenesis (Zhang et al., 2017; McGuire et al., 2019). The regulatory function of Tip60 on cancer is dependent on cancer types. As a tumor suppressor in most cancers such as breast cancer (Bassi et al., 2016), gastric cancer (Sakuraba et al., 2011) and advanced stage colorectal cancer (Mattera et al., 2009), downregulation of Tip60 leads to defective DNA repair inducing the accumulation of genetic mutations that can cause tumor progression (Bassi et al., 2016). On the contrary, Tip60 promotes prostate cancer progression via acetylation of androgen receptor (AR) to augment AR signaling (Halkidou et al., 2003; Shiota et al., 2010; Coffey et al., 2012).

Tip60 was found to be essential for the survival of thymic and peripheral Treg cells (Xiao et al., 2014). Overexpression of Tip60 promoted FOXP3-mediated transcriptional repression, suggesting that enhancing its function would alter T cell-mediated transcriptional inhibition (Li et al., 2007). For tumor immunity, studies have shown that Tip60 plays a critical role in fostering acetylation, dimerization and function in Treg cells, leading to tumor suppression. Accordingly, targeting ubiquitin-specific protease (Usp) 7, which controls Treg function primarily by stabilizing expression and promoting multimerization of Tip60 and Foxp3, limits tumor progression (Wang et al., 2016).

### HAT1

Histone acetyltransferase 1 (HAT1), also known as KAT1, was the first identified HAT (Poziello et al., 2021), and is a type of B histone acetyltransferase responsible for acetylating newly synthesized histones (Yang G. et al., 2021). As an oncogene, HAT1 is overexpressed and related to poor prognosis in a variety of solid tumors, such as HCCs (Jin et al., 2017), nasopharyngeal carcinoma (Miao et al., 2018), and pancreatic cancer (Fan et al., 2019), and can be a therapeutic target (Carafa et al., 2018). This may be due to the fact that HAT1 is involved in chromatin assembly and DNA damage repair, and its alterations can induce tumor development, invasion and metastasis (Poziello et al., 2021). HAT1 is also a transcription factor that upregulates the expression of various genes such as Bcl2L12 and Fas, and promotes cancer cell proliferation (Fan et al., 2019).

Despite being the first HAT to be discovered, HAT1 is still one of the least studied enzymes of its class (Poziello et al., 2021). In pancreatic cancer, HAT1 was shown to function as an important regulator in cancer immunity through transcriptional upregulation of PD-L1 in tumor cells, indicating that HAT1 could be used as a novel diagnostic and prognostic marker in immune checkpoint blockade therapy (Fan et al., 2019).

## Histone Deacetylases in Tumor Immunity Regulation

**FIGURE 1:**
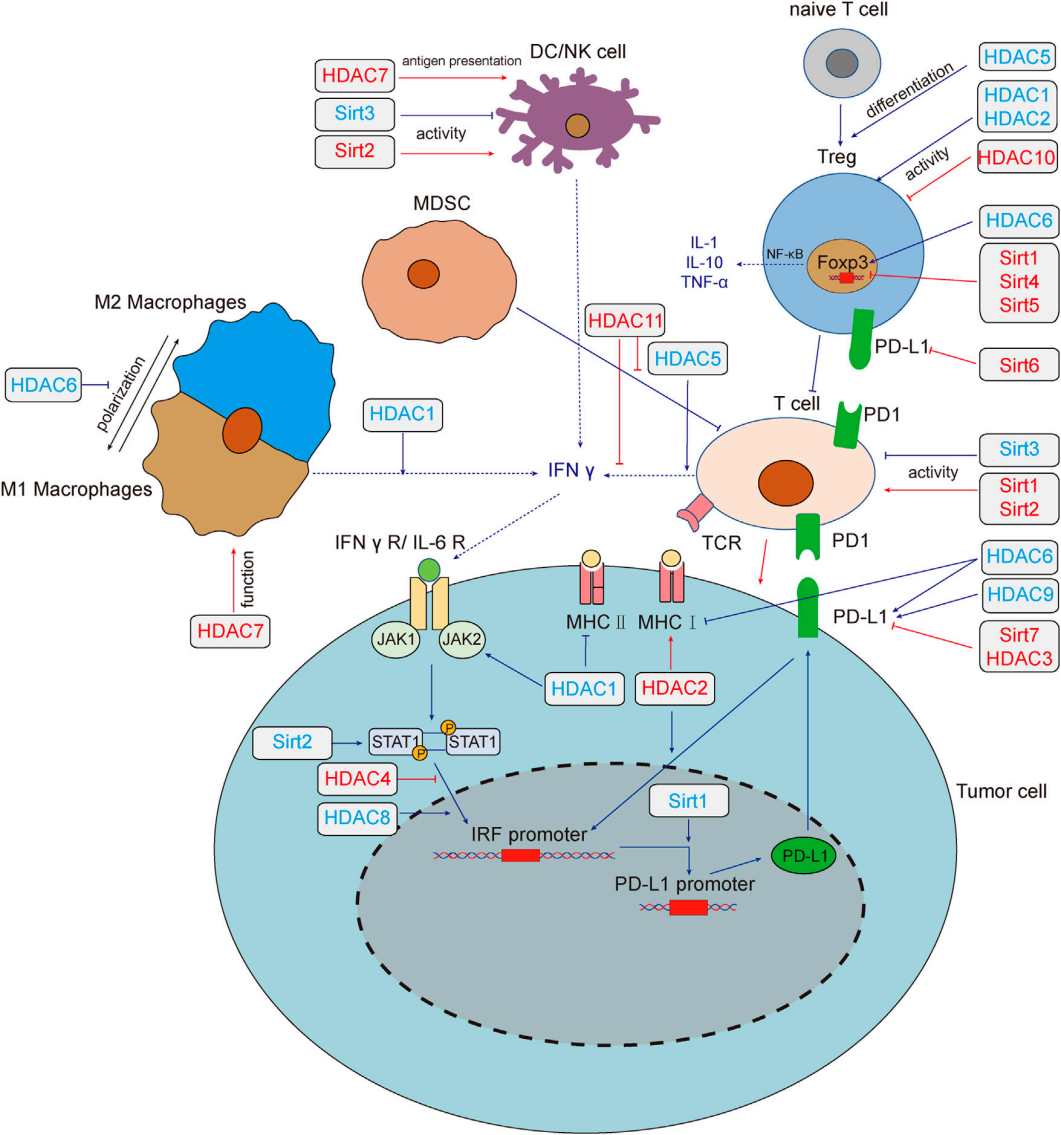
HADCs regulate tumor immunity and tumor immune microenvironment. Deacetylase regulates tumor immune response through multiple pathways. HDAC1,3,4,6,8,9 and Sirt1,2,6,7 regulate the expression of PD-L1 by regulating the IFN signaling pathway. HDAC1,2,6 regulates the expression of MHC, which affect the recognition of tumor cells by T cells. Sirt1, 2, 3 affect tumor immunity by changing the activity of T cells, HDAC1, 2, 10 could affect the function of Treg cells, and HDAC5 promotes the differentiation of naive T cells into Treg cells. Sirt2,3 could affect the activity of NK cells. HDAC7 both enhances the anti-tumor function of macrophages and the antigen presentation function of DC cells. HDAC1, 5, 6, 11 and Sirt1, 4, 5 modulate the tumor microenvironment by affecting the secretion of cytokines. Red lines and text colors represent the promoting effects of tumor immunity, while blue represents the tumor immunosuppression effects. Dotted lines indicate the regulation and function of cytokines. Abbreviations: T cell, T lymphocyte; NK, natural killer cell; DC, dendritic cells; MDSC, myeloid-derived suppressor cell; IFN-γ, interferon-γ; IFNγ R, interferon-γ receptor; IL, interleukin; JAK, Janus kinase; STAT, signal transducer and activator of transcription; TNF-α, tumor necrosis factor-α; Treg, regulatory T cell; IRF, interferon regulatory factor; PD-L1, programmed death-ligand 1; MHC, major histocompatibility complex; Sirt, sirtuins; HDAC, histone deacetylases.

Based on the homologies between HDAC and yeast deacetylases, HDACs can be divided into four sub-classes: Class I HDACs (HDAC1, 2, 3 and 8), which are similar to yeast Rpd3, are widely expressed *in vivo* and mainly located in the nucleus. Class II HDACs (HDAC4, 5, 6, 7, 9 and 10) have been studied that expressed in the cytoplasm and nucleus and distributed specifically in tissues. Class III HDACs (Sirt1-7) are similar to yeast Sir2, and its enzyme activity requires NAD+. Class IV HDAC (HDAC11) is homologous to yeast Rpd3 and Hda1. (Li and Seto, 2016). Studies have reported that more than 75% of human cancer tissues possess highly expressed class I HDACs (Nakagawa et al., 2007), besides, high expression of HDACs and low acetylation of histones are common in cancer cells (Cacan et al., 2014; Cacan, 2016; Alqinyah et al., 2017 ). The regulatory role of HDACs on tumor immunity was summarized in Figure 1.

### Histone Deacetylase Family

The HDAC family is a histone deacetylase subfamily that contains HDAC1-11, whose activity is independent of NAD+. It has been observed in multiple cancers that HDACs promotes the proliferation of cancer cells by reducing the expression of the cyclin-dependent kinase inhibitor p21 or TGF-β, in addition, Class I HDACs can also promote cancer cell metastasis by inhibiting the expression of E-cadherin (Glozak and Seto, 2007).

HDAC family plays an important regulatory role in immune cells via its histone deacetylation activity. For instance, the effects of HDACs have been reflected in all aspects of T cells, including T cell development, peripheral immature T cell pool formation, T cell activation and differentiation into effector T cells, activation of regulatory T (Treg) cells, secretion of cytokines, and T cell immune function (Kumari et al., 2013; Cacan, 2017; Ellmeier and Seiser, 2018). Besides, ample evidences have indicated that HDACs regulate the macrophage development, differentiation, polarization, and activation through a variety of signal pathways (Mohammadi et al., 2018).

In addition, HDAC also participates in immunomodulatory networks in cancer cells such as STAT and NF-κB, which not only regulate the expression of molecules in the signal pathway but also controls the nucleus translocation and degradation of STAT and NF-κB signal molecules (Villagra et al., 2010). Target HDACs can increase the expression of antigen presentation molecules such as MHC I, MHC II, CD40 or promote their translocation (Maeda et al., 2000; Khan et al., 2008). HDAC inhibitors can also increase the expression of PD-L1 in tumor cells, and the combination of HDAC inhibitors and PD-1 blockers can delay tumor progression and improve survival rate (Woods et al., 2015).

#### HDAC1

HDAC1, belonging to Class I, regulates N-terminal lysine residue deacetylation of histones to regulate gene transcription, cell cycle, growth, and apoptosis (Willis-Martinez et al., 2010). HDAC1 were significantly up-regulated in gastric cancer and could promote tumorigenesis and inhibit apoptosis (Yu et al., 2019). In addition, several recent studies have shown that HDAC1 inhibition is beneficial to the therapy of cancer, thus highlighting the cancer promoting function of HDAC1.

HDAC1/2 are required for normal T-cell development (Dovey et al., 2013), and HDAC1 regulates T cell-mediated autoimmunity by regulating CD4+T cells trafficking (Hamminger et al., 2021). Inhibition of HDAC1 promoted acetylation of histone H3/H4 in IFN-β1 promoter, and enhanced phosphorylation of interferon regulatory factor (IRF) 3 and its binding to the IFN-β promoter, which could lead to an anti-tumor immune response of macrophages microenvironment (Mounce et al., 2014; Meng et al., 2016). In addition, the role of HDAC1 in immune cells polarization has been highlighted. Several studies have shown that HDAC1 directly up-regulating the expression of miR-146a in macrophages, which induces tumor associated macrophages (TAMs) to adopt the M1-like phenotype. Besides, HDAC1 inhibition could promote the shift of microglia from M1 to M2 (Gao et al., 2015; Ji et al., 2019).

Mounting evidence shows that HDAC1 could regulate the expression of immune checkpoint molecules. HDAC1 inhibition could suppress the expression of PD-L1 induced by the IFN-γ signal pathway via down-regulating nucleus translocation of JAK2-mediated STAT1 in gastric cancer cells (Deng et al., 2018). In addition, the inhibition of HDAC1 could upregulate membrane expression of MHCⅡin cervical cancer and glioblastoma multiforme cells, and enhance the expression of CD1d in NSCLC cells (Yang et al., 2012; Wijdeven et al., 2018). HDAC1 also affects tumor immunity by regulating the secretion of cytokines. The CoREST complex, composed of HDAC1/2, LSD1, and scaffolding proteins Rcor1 and Rcor2, regulates a variety of immune and inflammatory responses. Targeting HDAC1/2 inhibits the binding of CoREST with IL-2 and the IFN-γ promoter, thereby promoting their expression, inhibiting the function of Treg, and enhancing anti-tumor immunity (Xiong et al., 2020).

#### HDAC2

Similar to HDAC1 in structure, HDAC2 has been shown vital in cardiac hypertrophy, Alzheimer’s disease, Parkinson’s and cancer (Kramer, 2009). HDAC2 inactivation can inhibit tumor cell growth and activate apoptosis by activating p53 and Bax (Jung et al., 2012). HDAC2 enhances proinflammatory cytokine production in LPS-stimulated macrophages by impairing the expression of c-Jun which are essential for the negative regulation of the inflammatory response (Wu et al., 2019) and inhibits the expression of plasminogen activator inhibitor 1 (PAI-1), TNF, and macrophage inflammatory protein-2 (MIP-2) in macrophage cells (Fang et al., 2018). Besides, studies have shown that HDAC2 enhances the infiltration of tumor lymphocytes and inhibits IL6 through its histone deacetylation function (Zhang et al., 2015; Xu et al., 2019).

P300-mediated PD-L1 acetylation at K263 inhibits the nucleus translocation of PD-L1, whereas HDAC2 deacetylate PD-L1 K263 and promote nucleus translocation of PD-L1. In the nucleus, PD-L1 interact with RelA and IRF proteins to form a positive feedback pathway to promote immune escape. Besides, treatment with HDAC2 inhibitors can also induce interferon type III related genes IL28A and IL28B, to activate STAT1 and increase the expression of MHC class I antigen presenting genes, thus achieving an improved immunotherapeutic effect (Gao et al., 2020).

#### HDAC3

HDAC3 plays an important role in apoptosis, cell progress, and DNA damage repair (Sarkar et al., 2020). In colorectal cancer and triple-negative breast cancer, the level of HDAC3 was upregulated. HDAC3 promotes the proliferation of colorectal cancer cells, HCC cells and glioma cells and inhibits the apoptosis of prostate cancer cells (Tong et al., 2020).

HDAC3 inhibition was reported to inhibit lipopolysaccharides (LPS) induced cytokine secretion in monocytes and M1 macrophages (Ghiboub et al., 2020). HDAC3 was reported to reduce the ratios of CD4^+^ and CD8^+^ T cells infiltration in colorectal carcinoma through upregulating B7x expression, which is related to a poor prognosis (Li et al., 2020b). HDAC3 inhibits the cytotoxicity of CD8+T cells and recognition ability of NK cells in melanoma cells by inhibiting genes encoding necessary cytotoxic proteins and key transcription factors (Fiegler et al., 2013; Tay et al., 2020).

Interestingly, depend on the cancer type, there are opposite reports on the regulation of PD-L1 by HDAC3. Studies have found that expression of PD-L1 is negatively correlated with HDAC3, suggesting that HDAC3 is a key inhibitor of PD-L1 transcription. Drugs targeting HDAC3 like resveratrol and pioglitazone upregulate PD-L1 expression in NSCLC, breast and colorectal cancer (Lucas et al., 2018; Wang H. et al., 2020). In B-cell lymphoma, HDAC3 is recruited to the PD-L1 promoter by the transcriptional inhibitor BCL6. In addition, HDAC3 inhibitors can also indirectly reduce the level of DNA methyltransferase 1 protein and activate PD-L1 transcription. HDAC3 inhibition combined with anti-PD-1/PD-L1 therapy can significantly improve the efficacy of B-cell lymphoma treatment (Deng et al., 2019). However, one study showed that the HDAC3/STAT3 pathway transcriptionally regulates PD-L1 expression in pancreatic ductal adenocarcinoma, and HDAC3 inhibitors reduce the protein and mRNA levels of PD-L1 in pancreatic cancer cells (Hu et al., 2019).

#### HDAC4

HDAC4, in class II, plays an important role in cell cycle progression and developmental events (Wang et al., 2014). HDAC4 was inhibited by miR-155 in human diffuse large B cell lymphoma (DLBCL) cells, resulting in up-regulation of downstream genes and induction of uncontrolled cell proliferation (Sandhu et al., 2012).

As an important signal pathway regulating tumor immunity, IFN signal promotes the phosphorylation of STAT and binds to the transcription factor interferon regulatory factor1 (IRF1) which promotes expression of PD-L1 (Kalbasi and Ribas, 2020). Although there is no direct report on the relationship between HDAC4 and tumor immunity, studies believe that HDAC4 can affect IFN signal. HDAC4 was reported to form a trimer with liver X receptor (LXR)α and pSTAT1, which block the binding of pSTAT1 to the promoter and inhibit the expression of d IRF1, TNF-α, and IL-6 in brain astrocytes (Lee et al., 2009). In cervical cancer cell line, HDAC4 was reported to promote type I interferon signaling via recruiting STAT2 to IFN-α–stimulated promoters (Lu et al., 2019).

#### HDAC5

HDAC5 is highly expressed in many tumors such as NSCLC, and melanoma but lowly expressed in gastric cancer (Yang J. et al., 2021). Several studies have shown that HDAC5 enhance the invasion and metastasis of neuroblastoma, pancreatic cancer and lung cancer. In addition, HDAC5 also inhibits the proliferation of tumor cells, which may be mediated by TGF-β (Yang J. et al., 2021).

HDAC5 has been reported to repress the production of proinflammatory cytokine in macrophages via TNF-α signaling (Li et al., 2017) and play an inhibitory role in regulating tumor microenvironments. HDAC5 deficient CD4+T cells lack the ability to transform into Tregs, while CD8+T cells impairs the ability to produce IFN- *γ* without HDAC5, which may offset the immune benefit resulting from decreased Treg function (Xiao et al., 2016). HDAC5-driven escape tumors exhibit a significant transition from neutrophils to macrophages. HDAC5 inhibits suppressor of cytokine signaling 3 (SOCS3), which leads to an increase of C-C motif chemokine ligand 2 (CCL2), recruits CCR2+ macrophages, promotes macrophages to reaggregate into tumor microenvironments, and promotes tumor recurrence (Cheng et al., 2014a; Hou et al., 2020).

#### HDAC6

HDAC6 protein is related to tumorigenesis, cell survival and metastasis of cancer cells (Aldana-Masangkay and Sakamoto, 2011). In breast cancer, HDAC6 promotes cell movement by acting on the nonhistone substrates, which enhance tumor cell movement, metastasis, and invasion. In addition, the expression of HDAC6 is closely related to endocytosis, and inhibits EGFR transport and degradation through α-tubulin deacetylation, thus activating cell proliferation through the downstream pathway of EGFR in NSCLC (Li et al., 2018).

HDAC6 and STAT3 form a complex and are recruited together to the immunosuppressive cytokine IL-10 promoter in antigen-presenting cells (APCs) to promote immune tolerance (Cheng et al., 2014b). In addition, NextA, a selective HDAC6 inhibitor, was found to increase the proportion of M1/M2 macrophages in tumor microenvironments, promoting IFN-γ and IL-2 levels and transformation of tumor microenvironments from “cold” to “hot”, thus enhancing the efficacy of immune checkpoint blocking therapy (Knox et al., 2019b).

HDAC6 has been also reported the two opposite regulation of PD-L1 in different tumor types. Several studies have reported that inhibition of HDAC6 decreases expression of PD-L1 in glioblastoma and melanoma. Selective inhibition of HDAC6 reduces the immunosuppressive activity of PD-L1 and leads to the recovery of host antitumor activity. A study suggested these effects may be mediated by recruitment and activation of STAT3 (M et al., 2016; Liu JR. et al., 2019). However, another study showed targeting HDAC6 can increase expression of PD-L1 and promote immunotherapy efficacy. Meanwhile, HDAC6 binds to cytoplasmic Foxo1 at K242 and deacetylates Foxo1 to weak the stability of Foxo1 and inhibit nucleus translocation, which limits IL-17–producing helper T (TH17) pathogenicity and antitumor effect in hepatocellular carcinoma (Qiu et al., 2020). Targeting HDAC6 in melanoma can up-regulate the expression of tumor-associated antigens and MHC-I molecules leading to enhance anti-tumor immunity (Woan et al., 2015).

#### HDAC7, 8, 9, 10, 11

Although the studies of HDAC7, 8, 9, 10 and 11 in tumorigenesis and progress have been widely reported, there is still a lack of research on their role in tumor immunity. Thereby, we summarize and describe the function of these enzymes in this part. HDAC7, 9, 10 belongs to the class Ⅱ HDACs, HDAC8 belongs to the class I HDACs, and HDAC11 is the only class IV HDAC, all of them affect the initial and progress of tumors in different ways. HDAC7 is considered a regulator of apoptosis in developing thymocytes (Dequiedt et al., 2003) and promoted cell proliferation through regulation of c-myc (Zhu et al., 2011). HDAC8 has been reported to promote the proliferation of hepatocellular carcinoma and inhibit apoptosis. On the contrary, targeting of HDAC8 inhibits the proliferation of lung cancer cell lines (Chakrabarti et al., 2015). HDAC9 is upregulated in various tumors such as glioblastoma, medulloblastoma (Yang C. et al., 2021). HDAC10 function as a tumor suppressor in stem-like lung adenocarcinoma and has been shown to interact with HDAC2 (Fischer et al., 2002; Li et al., 2020c). Notably, HDAC11 as the smallest HDAC isotype possesses very low deacetylase activity. Studies have shown that HDAC11 is highly expressed in prostate cancer, ovarian cancer, breast cancer, and NSCLC. Besides, targeting HDAC11 can enhance chemosensitivity (Liu SS. et al., 2020; Nunez-Alvarez and Suelves, 2021).

HDAC7, 8, 9, 10, 11 regulates immune function via affecting the polarization, antigen presentation, infiltration and activation of immune cells via various mechanisms. Previous studies have shown that HDAC7 and HDAC9 are involved in immune response mediated by immune effector cells. HDAC7 blocks the induction of genes that involved in macrophage immunity, phagocytosis, inflammation and cytokine production (Barneda-Zahonero et al., 2013). Besides, in uterine macrophages, HDAC9 deficiency promotes M2 macrophage polarization (Liu et al., 2021). HDAC9 also enhance immune response via enhancing dendritic cell antigen presentation and CD8+T cell TME infiltration (Ning et al., 2020). In addition, HDAC10 and HDAC11 promote the inhibition of immune response by immune regulatory cells. HDAC10 deletion Treg exhibited a stronger immune inhibitory effect and represses inflammation after intracerebral hemorrhage (Dahiya et al., 2020). MDSCs without HDAC11 exhibit stronger inhibitory activity against CD8+T cells via inducing high level of immunosuppressive enzymes expressed in CD8+T cells (Chen et al., 2021).

In addition, the secretion of cytokines is also an important apparent regulatory function of these enzymes. HADC7 represses the production of IFN-γ and promotes CD4^+^ T cells proliferation, as well as increasing the expression of the proinflammatory cytokine IL-1β in macrophage (Myers et al., 2017; Das Gupta et al., 2020). Similarly, in T cells, HDAC11 knockout increases the expression of transcription factors Eomesodermin and Tbx21, which reduce the production of inflammatory cytokines and increase the expression of IFN-γ (Woods et al., 2017). Besides, HDAC11 can bind to the promoter of IL-10 to inhibit IL-10 expression and induce inflammatory antigen-presenting cells to activate primordial antigen-specific CD4+T cells (Villagra et al., 2009).

In terms of tumor immunity, HDAC8, 9, and 10 have been reported to be related to the expression of immune checkpoint. HDAC8 can promote tumor immunity by inhibiting the expression of PD-L1. In melanoma cells, HDAC8 competitively inhibits the transcription factor to bind the PD-L1 promoter, thus inhibiting the expression of PD-L1 (Wang YF. et al., 2018). However, opposite studies have shown that HDAC8 inhibition increases the expression of NKG2D ligand in glioma cells which enhanced the recognition ability and cytotoxicity of NK cells, and activates immune cells in hepatocellular carcinoma resulting in an effective and lasting response to ICB (Yang W. et al., 2021; Mormino et al., 2021). On the contrary, HDAC9 and HDAC10 are opposite to HDAC8 in up-regulating PD-L1. One study has suggested that HDAC9 can significantly promote infiltration of immune cells and increase expression of immunological molecules such as PD-L1, CTLA4 and LAG3 in clear cell renal cell carcinoma (Fu Y. et al., 2020). In addition, the expression of HDAC10 in NSCLC was positively correlated with the expression of PD-L1, and the level of PD-L1 is significantly higher than paracancerous tissues, indicating a poor prognosis (Liu X. et al., 2020). Besides, HDAC10 inhibits NK cell-mediated antitumor immunity in hepatocellular carcinoma via recruiting EZH2 to block the CXCL10 promoter (Bugide et al., 2021).

### Sirtuin Family

The mammalian Sirtuin protein family is a homologue of Sir2, which can regulate various processes in mammalian cells and play a crucial part in regulating aging, metabolism, gene transcription, and stress responses (such as neurodegeneration, diabetes, cardiovascular disease and many types of cancer) (Michan and Sinclair, 2007). Sirtuins plays an indispensable role in tumorigenesis and development through cellular effects to genomic instability (regulation of cell cycle, DNA repair, cell survival and apoptosis), such as modulating cancer-related metabolism and changing the tumor microenvironment. There are few studies available. (Palmirotta et al., 2016).

#### Sirt1

Sirt1 (Sirtuin 1) mainly locates in the nucleus, and can removes acetyl groups from proteins. Sirt1 can inhibit transcription by directly deacetylating histones H1 lys26, H3 lys9、lys14, and H4 lys16 and by recruiting other ribozymes to chromatin to promote histone and DNA methylation changes that regulate chromatin function (Vaquero et al., 2004).

At present, the research of Sirt1 in tumor immunity is gradually in-depth. In T cells, Sirt1 enhances the activity of Foxo1 by deacetylating Foxo1, and promotes the expression of *Klf2* and *Ccr7* genes, thereby improving the anti-tumor immune response of T cells (Chatterjee et al., 2018). Additionally, the deacetylation of FoxP3 induced by Sirt1 is critical to the immunosuppressive function of Treg, resulting in Foxp3 degradation and reduced Treg cell number and activity (van Loosdregt et al., 2010). Sirt1 could enhance the tumor killing ability of macrophages, and deacetylate K310 of the p65 subunit in NF-κB, thereby increasing the infiltration of CD8+T cells in tumors or increasing the expression of CXCL10, so as to recruit NK cells and macrophages into tumor microenvironments (Yu et al., 2016a; Yu et al., 2016b; Zhou et al., 2019; Ye et al., 2020). Sirt1 can enhance the activity of NF-κB by promoting the phosphorylation of p65 and IκB, thereby promoting the polarization of M1 in hepatocellular carcinoma (Zhou et al., 2019).

In tumor microenvironments, Sirt1 participates in the immune response by activating the pro-inflammatory pathway (Chen et al., 2015). Pharmacological activation of Sirt1 increases the stability of the transcription factor snail, enhances the binding of β-catenin/TCF to the PD-L1 promoter, and promotes the expression of PD-L1 in NSCLC (Yang M. et al., 2021). Further research is needed on the specific regulation details of Sirt1 in tumor microenvironment.

#### Sirt2

Sirt2 is the only Sirtuin protein found mainly in the cytoplasm, and it is also expressed in mitochondria and nucleus (Wang Y. et al., 2019). Sirt2 plays an important role in cell differentiation, senescence, infection, inflammation, oxidative stress, and autophagy by regulating the function of important oncogenes, such as Myc and KRAS (Chen et al., 2020). Although there is clear evidence of that Sirt2 is abnormally expressed in various tumors, its causal relationship with tumorigenesis is still unclear, and its effect on the development of different kinds of tumors and its molecular mechanism are still contested (Song et al., 2016; Wang Y. et al., 2019).

Studies have found that the level of Sirt2 is positively correlated with CD8^+^ effector memory T (TEM) cells in peripheral T lymphocytes from breast cancer patients. Sirt2 can inhibits GSK3 β acetylation in CD8+T cells by promoting aerobic oxidation, which promotes the differentiation of CD8+T cells into TEM (Jiang et al., 2020). In chemically induced hepatocellular carcinoma mice, it has been found that sirt2 was induced in the immune microenvironment of hepatoma cells to enhance the tumor-killing activity of NK cells by promoting mitogen-activated protein kinase (MAPK) in activated NK cells (Chen M. et al., 2019). In addition, Sirt2 can regulate IFN-driven gene transcription by regulating the phosphorylation of Ser-727 on IFN I-dependent STAT1 through deacetylation of CDK9 Lys48 (Kosciuczuk et al., 2019).

#### Sirt3

Sirt3 plays an important role in mitochondrial function, aging, and carcinogenesis by targeting a series of key regulatory factors and their related pathways in tumors to regulate cell death (Chen et al., 2014). A study has shown that Sirt3 deacetylates NLRC4 to promote its activation, thereby promoting the activation of inflammasomes and mediating the production of the pro-inflammatory cytokine IL-1β in macrophages (Guan et al., 2021).

Blocking Sirt3 and Sirt6 can inhibit the activation of RIPK3 and MLKL in prostate cancer cells, thus enhancing necrotizing apoptosis and promoting infiltration of CD4+T cells, macrophages and neutrophils, so as to inhibit the progression of cancer (Fu W. et al., 2020).

#### Sirt4

Sirt4 is located in mitochondria and plays an important role in cellular metabolism and tumor biology (Tomaselli et al., 2020). It is mostly regarded as a tumor suppressor gene, and its expression is low in breast, gastric, and colon cancers (Huang and Zhu, 2018). Sirt4 can inhibit FoxP3, anti-inflammatory cytokines IL-10, TGFβ and AMPK signal in Treg cells, which impairs their anti-inflammatory function (Lin et al., 2019).

#### Sirt5

Sirt5 promotes cancer cell proliferation by targeting a variety of metabolic enzymes including GLS, SHMT2, and PKM2 (Abril et al., 2021). It can maintain histone acetylation and methylation levels in melanoma, thereby promoting the corresponding expression of MITF and c-myc (Milling and Edgar, 2019). It is reported that Sirt5 affects the tumor immune microenvironment through its succinylation function, which is negatively correlated with Treg infiltration in clear cell renal cell carcinoma (Lu et al., 2021).

#### Sirt6

Sirt6 is a NAD + -dependent histone H3K9 deacetylase that prevents genomic instability, maintains telomere integrity, and regulates metabolic homeostasis and DNA repair (Desantis et al., 2018). It inhibits IGF-Akt and NF-κβ signal transduction by the deacetylation of H3K9 (Sundaresan et al., 2012). High Sirt6 expression levels are observed in the immune system. It may be a possibly a negative regulator of immune cell function and metabolism, which related to neutrophil inactivation and increased the polarization to M2 macrophages (Pillai and Gupta, 2021).

Induction of Sirt6 expression in 4T1 cells inhibits the activation of NF-κB and suppresses the transcription of PD-L1 (Song et al., 2020), which suppresses the proliferation and transcription of PD-L1 in Treg cells (Vanamee and Faustman, 2017). In addition, overexpression of Sirt6 in pancreatic cancer cells increases the production of TNF and IL8 which has nothing to do with the activation of NF-κB, thus leading to pro-inflammatory and pro-angiogenic phenotype (Bauer et al., 2012).

#### Sirt7

Sirt7 plays an important role in ribosomal biogenesis, cellular stress response, genomic stability, metabolic regulation, aging, and cancer (Ford et al., 2006; Blank and Grummt, 2017; Tang et al., 2021). Compared with other nucleus-localized Sirts, Sirt7 exhibits weaker deacetylase activity. The enzyme activity of Sirt7 targets acetylated H3K18 and succinylated H3K122 (Li L. et al., 2016).

Sirt7 is high expressed in breast cancer, which is an indicator of poor prognosis, and the expression of Sirt7 is positively correlated with the expression of IRF5 and PD1, which increases M1 macrophages, and depletes T cells in the immune environment of breast cancer (Huo et al., 2020). Sirt7 knockout hepatocellular carcinoma cells expressed higher levels of PD-L1 by inhibiting the deacetylation of MEDF2D and reduced T cell infiltration and activation. Moreover, Sirt7 inhibition combined with PD1 blocking therapy can significantly improve the efficacy of hepatocellular carcinoma (Xiang et al., 2020).

## Targeting Acetylation in Tumor Immunotherapy

Immune checkpoint blockades such as anti-PD-1/PD-L1 and anti-CTLA4 have shown promising effects in cancer treatment. However, only few patients benefit from immunotherapy. The combinations of immunotherapy and acetylation-modified drugs (HAT inhibitors and HDAC inhibitors) have attracted more attention in tumor treatment due to the role of acetylases in the regulation of tumor and tumor immunity.

Currently available HAT inhibitors are primarily target CBP and p300, including E7386 (Yamada et al., 2021), PRI-724 (Osawa et al., 2015; Osawa et al., 2019), and A485 (Lasko et al., 2017; Liu J. et al., 2020), which have shown promising effects in improving anti-PD1 immunotherapy. Among them, E7386 (Yamada et al., 2021) and PRI-724 (Osawa et al., 2015; Osawa et al., 2019) inhibit the interaction between CBP and/or β-catenin, which can increase the infiltration of CD8^+^ T cells. A485 (Lasko et al., 2017; Liu J. et al., 2020) directly targets p300/CBP and inhibits the secretion of exosomal PD-L1.

Correspondingly, partial HDAC inhibitors can also modulate immunotherapies. Some drugs enhance tumor immunity by up regulating PD-L1 in tumor cells, such as Panobinostat(LBH589) (Atadja, 2009; Woods et al., 2015), Chidamide (CS055/HBI-8000) (Ning et al., 2012; Yan et al., 2020; Que et al., 2021), Trichostatin-A (TSA) (Li et al., 2021) and ACY738 (Regna et al., 2016; Maharaj et al., 2020). Other drugs can achieve antitumor immunity by increasing the infiltration of cytotoxic cells, including Nexturastat A (Knox et al., 2019a), Mocetinostat (Boumber et al., 2011; Briere et al., 2018), Domatinostat (4SC-202) (Bretz et al., 2019), Chidamide (CS055/HBI-8000) (Ning et al., 2012; Yan et al., 2020; Que et al., 2021), Valproic acid (VPA) (Xie et al., 2018; Adeshakin et al., 2020), Tacedinaline (CI994) (Berger et al., 1990; el-Beltagi et al., 1993; LoRusso et al., 1996; Burke et al., 2020), CXD10 (Eyre et al., 2019; Blaszczak et al., 2021), Nexturastat A (Knox et al., 2019a), AR42 (Booth et al., 2017) and Trichostatin-A (TSA) (Li et al., 2021), which can improve the infiltration of macrophages, NK cells, and neutrophils. In addition, Mocetinostat (Boumber et al., 2011; Briere et al., 2018), Chidamide (CS055/HBI-8000) (Ning et al., 2012; Yan et al., 2020; Que et al., 2021), Valproic acid (VPA) (Xie et al., 2018; Adeshakin et al., 2020), Trichostatin-A (Li et al., 2021) and Belinostat (PDX101) (Atadja, 2009; Llopiz et al., 2019) can also play an immune regulatory role by reducing the infiltration of immunosuppressive cells such as myeloid-derived suppressor cell (MDSC) and T-regulatory cells (Tregs). Finally, ACY738 (Regna et al., 2016; Maharaj et al., 2020) enhances MHC-restricted antigen presentation by upregulating tumor cell MHC-I expression.

## Conclusion

Over the last few decades, we have recognized acetylation plays an important role in the regulation of protein function, chromatin structure and gene expression. Research into this field has covered metabolism, immunity, cell cycle, DNA damage repair, apoptosis and autophagy. New discoveries reveal the evidence that acetylation may also influence the tumor immunity through several pathways including regulating the expression and function of immune checkpoint molecules and antigens in tumor cells, as well as processes such as infiltration, secretion of cytokines and antigen presentation by immune cells. Emerging interest in acetylation research has been centered on the use of HAT or HDAC inhibitors that have shown great potential for improving immunotherapeutic outcomes. Further studies should focus on safety and the best way to combine these drugs with various immunotherapies to conquer cancer in the future.

## Glossary

HAT: histone acetyltransferase
HDAC: histone deacetylase
ICs: immune checkpoints
PD-1: programmed cell death protein 1
PD-L1: programmed death-ligand 1
CTLA-4: cytotoxic T lymphocyte-associated protein 4
LAG: lymphocyte activation gene
ACT: adoptive T-cell therapy
ICB: immune checkpoint blockade
MHC: major histocompatibility class
CTLs: cytotoxic T cells
IL: interleukin
Tregs: regulatory T cells
IRF: interferon regulatory factor
IFN: interferon
TNF: tumor necrosis factor
NK: natural killer cell
Sirt: Sirtuin
NF- κ B: nucleus factor κ-light-chain enhancer of activated B cells
CXCL: C-X-C motif chemokine ligand
EMT: epithelial-mesenchymal transition
MDSCs: myeloid-derived suppressor cells
NSCLC: Non-small-cell carcinoma
Lys: lysine
Ser: serine
JAK: Janus kinase
STAT: signal transducer and activator of transcription
TAM: tumor associated macrophage
CoREST: repressor element 1 silencing transcription factor corepressor
DLBCL: diffuse large B cell lymphoma
Foxo: forkhead box O
NCOR2: nucleus receptor co-repressor 2
EZH: enhancer of zeste homolog 2
APC: antigen-presenting cell
TEM: effector memory T
RIPK: receptor interacting protein kinase
MLKL: mixed lineage kinase domain like pseudokinase
TGF: transforming growth factor
AMPK: AMP-activated protein kinase
GBM: glioblastoma
